# Adiposity, Body Fat Distribution, and Risk of Major Stroke Types Among Adults in the United Kingdom

**DOI:** 10.1001/jamanetworkopen.2022.46613

**Published:** 2022-12-14

**Authors:** Preyanka Pillay, Sarah Lewington, Hannah Taylor, Ben Lacey, Jennifer Carter

**Affiliations:** 1 Clinical Trial Service Unit and Epidemiological Studies Unit, Nuffield Department of Population Health, Big Data Institute, University of Oxford, Oxford, United Kingdom; 2 Medical Research Council Population Health Research Unit, Clinical Trial Service Unit, Nuffield Department of Population Health, University of Oxford, Oxford, United Kingdom

## Abstract

**Importance:**

Uncertainty persists regarding the independent association of general and central adiposity with major stroke types.

**Objective:**

To determine the independent associations of general and central adiposity with risk of ischemic stroke, intracerebral hemorrhage, and subarachnoid hemorrhage.

**Design, Setting, and Participants:**

Between 2006 and 2010, adults without prior stroke at recruitment in England, Scotland, and Wales were recruited into the UK Biobank prospective cohort study and were followed up through linkage with electronic medical records. Data were analyzed from September 2021 to September 2022.

**Exposures:**

General adiposity (body mass index [BMI] calculated as weight in kilograms divided by height in meters squared) and central adiposity (waist circumference).

**Main Outcomes and Measures:**

Incident ischemic stroke, intracerebral hemorrhage, and subarachnoid hemorrhage as recorded through record linkage with electronic hospital records. Cox regression estimated adjusted hazard ratios (HRs) by stroke type.

**Results:**

Among 490 071 participants, the mean (SD) age was 56.5 (8.1) years, 267 579 (54.6%) were female, and 461 647 (94.2%) were White. During a median (IQR) of 12 (11.2-12.7) years follow-up, 7117 incident ischemic strokes, 1391 intracerebral hemorrhages, and 834 subarachnoid hemorrhages were identified. After mutual adjustment for waist circumference, BMI had no independent association with ischemic stroke (HR per 5-unit higher BMI, 1.04; 95% CI, 0.97-1.11), but was inversely associated with intracerebral hemorrhage (HR, 0.85; 95% CI, 0.74-0.96) and subarachnoid hemorrhage (HR, 0.82; 95% CI, 0.69-0.96). Waist circumference (adjusted for BMI) was positively associated with ischemic stroke (HR per 10-cm higher waist circumference, 1.19; 95% CI, 1.13-1.25) and intracerebral hemorrhage (HR, 1.17; 95% CI, 1.05-1.30), but was not associated with subarachnoid hemorrhage (HR, 1.07; 95% CI, 0.93-1.22).

**Conclusions and Relevance:**

In this large-scale prospective study, the independent and contrasting associations of BMI and waist circumference with stroke types suggests the importance of considering body fat distribution to stroke risk. Waist circumference was positively associated with the risk of ischemic stroke and intracerebral hemorrhage, while BMI was inversely associated with the risk of subarachnoid hemorrhage and intracerebral hemorrhage. This study also suggests that some adverse correlate of low BMI may be associated with an increased risk of intracerebral hemorrhage and subarachnoid hemorrhage, which warrants further investigation.

## Key Points

**Question:**

What are the independent associations of general adiposity (body mass index [BMI]) and central adiposity (waist circumference) with ischemic stroke, intracerebral hemorrhage, and subarachnoid hemorrhage?

**Findings:**

This cohort study of 490 071 adults in the UK found that, following mutual adjustment for BMI and waist circumference, BMI was not associated with ischemic stroke and was inversely associated with intracerebral and subarachnoid hemorrhage. Waist circumference was positively associated with ischemic stroke and intracerebral hemorrhage but not associated with subarachnoid hemorrhage.

**Meaning:**

These findings suggest that central and general adiposity have independent and contrasting associations with risk of different stroke types.

## Teaser

This cohort study evaluates the association of general and central adiposity with risk of major stroke types among adults in the United Kingdom.

## Introduction

In 2019, stroke was the second leading cause of death globally, estimated to be responsible for approximately 10% of all deaths.^1^ Of the 12.2 million incident strokes worldwide in 2019, approximately two-thirds were ischemic strokes, one-quarter were intracerebral hemorrhages (ICHs), and one-tenth were subarachnoid hemorrhages (SAHs).^1^ In the United Kingdom (UK), stroke incidence is increasing after a longstanding decline,^2^ possibly due to an increasing prevalence of major modifiable cardiovascular risk factors, including adiposity, blood pressure, dyslipidemia, and diabetes.^1^ However, the evidence regarding the associations of body mass index (BMI; calculated as weight in kilograms divided by height in meters squared) and other measures of adiposity with different stroke types is limited.^3^

General adiposity (BMI) and central adiposity (waist circumference) have well-established, positive associations with ischemic stroke.^4,5^ However, uncertainty remains regarding their associations with ICH and SAH, and with the independent associations of general and central adiposity with each of the stroke types.^6^ Many studies have not used neuroimaging-confirmed diagnoses, without which the reliability of diagnosing stroke types is reduced, and most have had insufficient events of each stroke type (particularly for ICH and SAH) to assess reliably the strength of the association.^7^ As the pathophysiology of ischemic stroke, ICH, and SAH are different,^8,9^ identifying independent associations of adiposity measures with stroke types, and evaluating by how much known cardiovascular risk factors mediate any associations, would enable a greater understanding of their causes and of the burden of stroke attributable to adiposity in the population. This is particularly important for hemorrhagic stroke as mortality rates and functional outcomes are worse than those for ischemic stroke,^10^ despite advances in treatment.^9^ We report the independent associations of BMI and waist circumference with risk of incident ischemic stroke, ICH, and SAH in a large-scale prospective cohort study of UK adults, which has both detailed measures of adiposity and neuroimaging-confirmed diagnoses of stroke types.

## Methods

Between 2006 to 2010, 9.2 million people aged between 40 to 69 years and registered with the UK’s National Health Service (NHS) were invited to attend a UK Biobank assessment center in England, Scotland, or Wales, and 502 678 people consented to join the UK Biobank prospective cohort study.^11^ At the baseline visit, sociodemographic, lifestyle, and other health-related data were collected using self-completed touchscreen questionnaires and verbal interviews. Physical measurements were then made, and biological samples (including blood, urine, and saliva) were taken for long-term storage. The first resurvey was performed on a subset of about 20 000 participants between 2012 to 2013 and involved a repeat of the baseline data collection, which enabled assessment of within-person variability of exposure measures. The UK Biobank received ethical approval from the North West Multicenter Research Ethics Committee. All participants provided written informed consent.^11^ Reporting of analyses and results followed the Strengthening the Reporting of Observational Studies in Epidemiology (STROBE) reporting guideline.^12^

Various anthropometric measures were collected at baseline and at resurvey.^13^ Standing height was measured to the nearest 0.1 cm using a Seca 240-cm height measure. Weight was measured to the nearest 0.1 kg using a Tanita BC418MA body composition analyzer or with standard scales if participants did not undergo body composition analysis.^13^ BMI was calculated by dividing the weight in kilograms by the height in meters squared. With participants standing and dressed in minimal clothing, waist circumference was measured on expiration using a 200-cm Seca tape at the narrowest part of the trunk (ie, the natural indent) or at the level of the umbilicus if the natural indent was not found.^13^ Exposures were measures of general (BMI) and central (waist circumference) adiposity.

To obtain data on fatal and nonfatal incident stroke events, participants were followed up by linkage to national electronic health databases, including Hospital Episode Statistics (HES) and death data of the Office for National Statistics. Stroke events were classified by the *International Statistical Classification of Diseases and Related Health Problems, Tenth Revision (ICD-10)*, and included SAH (*ICD-10* code I60), ICH (*ICD-10* code I61), ischemic stroke (*ICD-10* code I63), and unspecified stroke (*ICD-10* code I64).

### Statistical Analysis

Analyses excluded those who had withdrawn from the study at the time of analysis, those with missing or implausible adiposity measures, and those with prior stroke or transient ischemic attack at baseline (identified from self-reported history at recruitment or HES records). Approximately normally distributed continuous variables were expressed as mean (SD), and skewed variables are presented as median (IQR). Cox proportional hazards models, with age-at-risk as the time scale, were used to estimate hazard ratios (HRs) and 95% CIs for the association of adiposity measures with incident stroke types. Multivariable models were adjusted for sex, race (participants responded to a survey item with the following options: Asian, Black, Chinese, Mixed, White, and other ethnic groups combined), Townsend deprivation quintile (5 categories), education (4 categories), smoking status (never, previous, and current), and alcohol intake (never, previous, current intake ≤14 units/week, current intake >14 units/week, and current intake unspecified units). Race was included in this analysis as it may be related to different distributions of body fat and cardiovascular risk factors.

To investigate the shape of the associations, HRs were calculated for fifths of each adiposity measure and plotted against the mean at resurvey in each baseline-defined fifth; HRs were reported relative to the lowest group, and group-specific variances were used to calculate 95% CIs, which reflect the amount of information only in that single group.^14^ Regression dilution ratios were calculated with Spearman self-correlation coefficients between adiposity measurements at baseline (2006-2010) and first resurvey (2012-2013). The estimated log_e_ HRs (and their SEs) were divided by regression dilution ratios to obtain HRs for usual levels of adiposity measures. Effect modification by sex and age groups (35-59, 60-69, and ≥70 years) were assessed by including an interaction term in the multivariable model and performing a likelihood ratio test.

A semiquantitative estimate of mediation was evaluated with a likelihood ratio test that assessed, for each adiposity measure, the change in the likelihood ratio χ^2^ statistic for that adiposity measure following sequential adjustment for intermediate factors considered to lie on the spectrum of associations between adiposity and stroke types. Intermediate factors included well-established cardiovascular risk factors such as elevated blood pressure (systolic blood pressure [SBP] and antihypertensive medication use), dyslipidemia (low-density lipoprotein cholesterol and statin use), triglycerides, and glycated hemoglobin A_1c _(HbA_1c_). Other intermediate factors assessed included C-reactive protein, estimated glomerular filtration rate, alanine transaminase, and uric acid. To assess the independent associations of general (BMI) and central adiposity (waist circumference) with stroke types, the change in the likelihood ratio χ^2^ statistic was calculated for multivariable models before and after mutual adjustment for these adiposity measures.

The Cox proportional hazards assumption was tested by comparing the HRs in the first and second half of follow-up (there was no evidence of violation). Sensitivity analyses evaluated the association of waist-hip ratio (which is another measure of central adiposity) with stroke types, and the potential impact of reverse causality by excluding the first 5 years of follow-up and those with a history of ischemic heart disease, myocardial infarction, cancer, emphysema, diabetes, congestive cardiac failure, chronic kidney disease, or chronic liver disease. All *P* values reported are 2-sided, the level of significance is P < .05, and CIs are 95%. All statistical analyses were performed using Stata statistical software version 16 (StataCorp). Data were analyzed from September 2021 to September 2022.

## Results

Of 502 678 participants recruited, 490 071 remained in the analysis after excluding those who had withdrawn from the study, those with adiposity measures that were missing (3111 participants) or implausible (1341 participants), and those with a self-reported or HES record of stroke or transient ischemic attack before recruitment (3845 participants). Following exclusions, participants had a mean (SD) age of 56.5 (8.1) years, 267 579 (54.6%) were female, and 461 647 (94.2%) were White. Overall, the mean (SD) BMI and waist circumference were 27.3 (4.7) and 90.1 (13.2) cm, respectively. Those with a higher BMI tended to be of lower socioeconomic status (as measured by Townsend deprivation), lower prevalence of smoking, higher prevalence of diabetes, and higher mean SBP (Table). Baseline BMI and waist circumference measurements had a positive correlation (*r* = 0.81). The regression dilution ratios for BMI and waist circumference, calculated using baseline and resurvey data, were 0.94 for BMI and 0.86 for waist circumference.

**Table.** Baseline Characteristics of Participants in UK Biobank

Characteristics	Participants, No. (%)					
	Fifths of baseline BMI^a^					All (N = 490 071)
	15.0 to <23.5 (n = 98 020)	23.5 to <25.6 (n = 98 060)	25.6 to <27.8 (n = 97 974)	27.8 to <30.8 (n = 98 007)	≥30.8 (n = 98 010)	
Sociodemographic factors						
Age, mean (SD), y	55.2 (8.3)	56.4 (8.1)	56.9 (8.1)	57.1 (8.0)	56.7 (7.9)	56.5 (8.1)
Sex						
Female	69 398 (70.8)	54 521 (55.6)	46 440 (47.4)	44 103 (45.0)	54 102 (55.2)	267 579 (54.6)
Male	28 622 (29.2)	43 539 (44.4)	51 534 (52.6)	53 904 (55.0)	43 908 (44.8)	222 492 (45.4)
White ethnicity	92 825 (94.7)	92 765 (94.6)	92 487 (94.4)	92 127 (94.0)	91 541 (93.4)	461 647 (94.2)
University or professional qualification	55 185 (56.3)	54 129 (55.2)	52 416 (53.5)	50 768 (51.8)	48 711(49.7)	261 208 (53.3)
Townsend deprivation most deprived quintile	18 428 (18.8)	17 062 (17.4)	17 341 (17.7)	19 601 (20.0)	25 385 (25.9)	98 014 (20.0)
Medical history						
Hypertension^b^	38 522 (39.3)	48 834 (49.8)	56 041 (57.2)	63 215 (64.5)	71 449 (72.9)	283 751 (57.9)
Diabetes	1568 (1.6)	2353 (2.4)	3429 (3.5)	5684 (5.8)	11 761 (12.0)	24 994 (5.1)
Self-reported medication use						
Antihypertensive	8528 (8.7)	13 238 (13.5)	18 125 (18.5)	23 914 (24.4)	34 402 (35.1)	98 014 (20.0)
Statins	6861 (7.0)	11 473 (11.7)	15 970 (16.3)	20 385 (20.8)	25 973 (26.5)	80 862 (16.5)
Insulin	588 (0.6)	588 (0.6)	686 (0.7)	1078 (1.1)	2254 (2.3)	5391 (1.1)
Warfarin	490 (0.5)	588 (0.6)	784 (0.8)	980 (1.0)	1372 (1.4)	4411 (0.9)
Lifestyle behaviors						
Current smoker	11 762 (12.0)	10 198 (10.4)	9797(10.0)	9997 (10.2)	9409 (9.6)	50 967 (10.4)
Alcohol intake >14 U/wk	37 640 (38.4)	42 244 (44.1)	45 656 (46.6)	45 671 (46.6)	37 440 (38.2)	209 750 (42.8)
Clinical and biochemical measures^c^						
Systolic blood pressure, mean (SD), mm Hg	131.0 (18.7)	136.2 (18.5)	139.0 (18.1)	140.9 (17.9)	142.0 (17.7)	137.8 (18.6)
LDL cholesterol, mean (SD), mg/dL	131.3 (30.9)	139.0 (30.9)	139.0 (34.8)	139.0 (34.8)	135.1 (34.8)	139.0 (34.8)
Triglycerides, median (IQR), mg/dL	95.8 (72.8-131.3)	117.3 (85.9-165.1)	136.3 (97.6-193.2)	153.2 (109.0-217.7)	166.8 (120.2-232.7)	131.2 (92.5-189.9)
Glycated hemoglobin A_1c_, mean (SD), %	5.3 (2.6)	5.3 (2.6)	5.4 (2.7)	5.5 (2.8)	5.7 (3.0)	5.4 (2.7)
C-reactive protein, median (IQR), mg/dL	0.07 (0.04-0.14)	0.10 (0.05-0.19)	0.13 (0.07-0.24)	0.17 (0.09-0.31)	0.27 (0.15-0.50)	0.13 (0.07-0.27)
eGFR (CKD-EPI), mean (SD), mL/min/1.73 m	102.5 (26.3)	98.3 (24.8)	96.3 (24.4)	95.1 (26.2)	95.8 (27.6)	97.6 (26.0)
Alanine transaminase, median (IQR), U/L	16.4 (13.2-20.9)	18.4 (14.5-23.9)	20.5 (15.9-27.3)	22.7 (17.3-30.7)	24.6 (18.3-34.1)	20.1 (15.4-27.4)
Uric acid, mean (SD), mg/dL	5.0 (1.7)	5.0 (1.7)	5.0 (1.7)	5.0 (1.7)	6.7 (0.1)	5.0 (1.7)
Adiposity measures, mean (SD)						
Height, cm	167.4 (8.7)	168.7 (9.2)	169.3 (9.4)	169.3 (9.4)	167.6 (9.5)	168.5 (9.3)
Weight, kg	61.2 (7.7)	70.3 (8.0)	76.9 (8.7)	83.9 (9.6)	97.2 (13.8)	77.9 (15.7)
Body mass index	21.8 (1.4)	24.7 (0.6)	26.7 (0.6)	29.2 (0.8)	34.5 (3.5)	27.3 (4.7)
Waist circumference, cm	75.7 (7.4)	83.8 (7.5)	89.7 (7.7)	95.6 (8.0)	106.1 (10.7)	90.1 (13.2)

Abbreviations: CKD-EPI, Chronic Kidney Disease Epidemiology Collaboration; eGFR, estimated glomerular filtration rate; LDL, low-density lipoprotein.

SI conversion factors: To convert LDL cholesterol to millimoles per liter, multiply by 0.0259; triglycerides to millimoles per liter, multiply by 0.0113; hemoglobin A_1c_ to proportion of total hemoglobin, multiply by 0.01; C-reactive protein to milligrams per liter, multiply by 10; alanine transaminase to microkatal, multiply by 0.0167; uric acid to millimoles per liter, multiply by 0.0595.

^a^ Body mass index is calculated as weight in kilograms divided by height in meters squared.

^b^ Defined as self-reported hypertension diagnosis, or systolic blood pressure ≥140 mm Hg or diastolic blood pressure ≥90 mm Hg, at baseline.

^c^ Measured in a subset of 419 292 participants.

**Figure 1.**
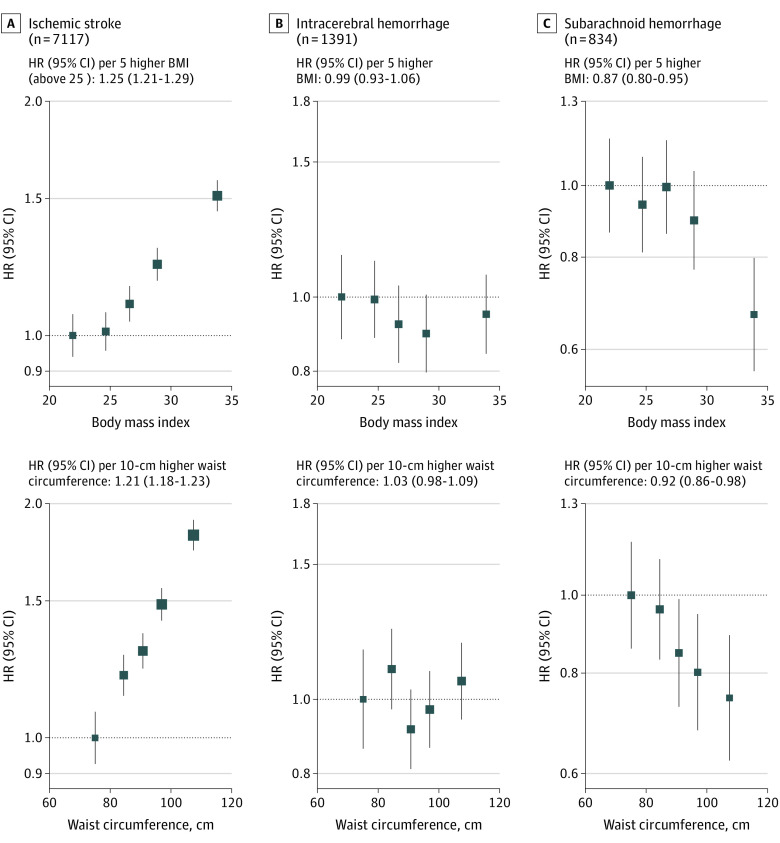
Hazard Ratios (HRs) and 95% CIs per Fifths and Specified Unit Increase in Adiposity Measures by Stroke Type Analyses adjusted for age, ethnicity, Townsend deprivation, education, smoking status, and alcohol intake. Group-specific estimates are plotted as squares, with the size of each square proportional to the amount of statistical information. Vertical lines represent group-specific 95% CIs.

During a median (IQR) follow-up of 12 (11.2-12.7) years, there were 7117 incident ischemic strokes, 1391 ICHs, and 834 SAHs. After adjustment for potential confounders, usual BMI had a log-linear positive association with ischemic stroke per 5-unit higher BMI above 25 (HR, 1.25; 95% CI, 1.21-1.29), no association with ICH (HR, 0.99; 95% CI, 0.93-1.06), and an inverse association with SAH (HR, 0.87; 95% CI, 0.80-0.95). Similarly, waist circumference had a log-linear positive association with ischemic stroke per 10-cm higher waist circumference (HR, 1.21; 95% CI, 1.18-1.23), no association with ICH (HR, 1.03; 95% CI, 0.98-1.09), and a log-linear inverse association with SAH (HR, 0.92; 95% CI, 0.86-0.98) (Figure 1). There was some evidence of effect modification by sex and age in these associations, most notably for ischemic stroke, which attenuated the association for both BMI and waist circumference at older ages (eFigure 1 in Supplement 1).

**Figure 2.**
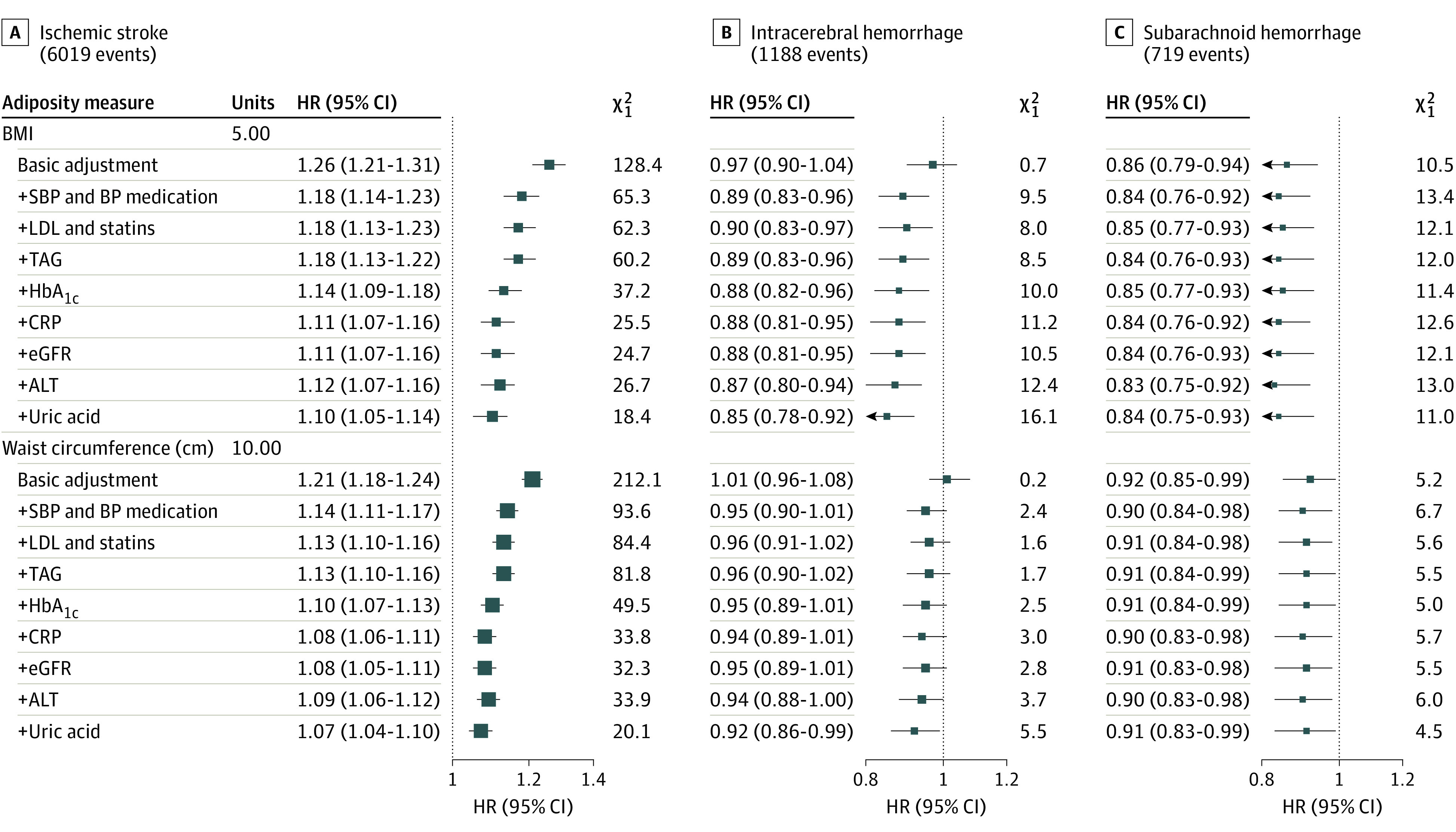
Hazard Ratios (HRs) and 95% CIs per Specified Unit Increase in Adiposity Measures With Sequential Adjustment Intermediate Factors, by Stroke Type Analyses performed in a subset of 419 292 people with data on all intermediate factors. Analyses adjusted for age, ethnicity, Townsend deprivation, education, smoking status, and alcohol intake. HRs are plotted as squares, with the size of each square proportional to the amount of statistical information. Horizontal lines represent 95% CIs. BMI with ischaemic stroke is only for the linear portion of BMI>25. ALT indicates alanine transaminase; BP, blood pressure; BMI, body mass index; CRP, C-reactive protein; eGFR, estimated glomerular filtration rate; HbA_1c_, glycated hemoglobin; LDL, low-density lipoprotein cholesterol; SBP, systolic blood pressure; TAG, triglycerides.

Approximately 80% of the positive associations of BMI and waist circumference with ischemic stroke were explained by a set of well-established cardiovascular risk factors (SBP, low-density lipoprotein cholesterol, triglycerides, and HbA_1c_), and associations were only minimally attenuated after further adjusting for C-reactive protein, estimated glomerular filtration rate, alanine transaminase, and uric acid (Figure 2). BMI became inversely associated with ICH following adjustment for SBP (HR, 0.89; 95% CI, 0.83-0.96), with further adjustment for other potential intermediate factors having little impact. However, associations of BMI and waist circumference with SAH were unchanged after adjusting for any measured intermediate factors (Figure 2).

**Figure 3.**
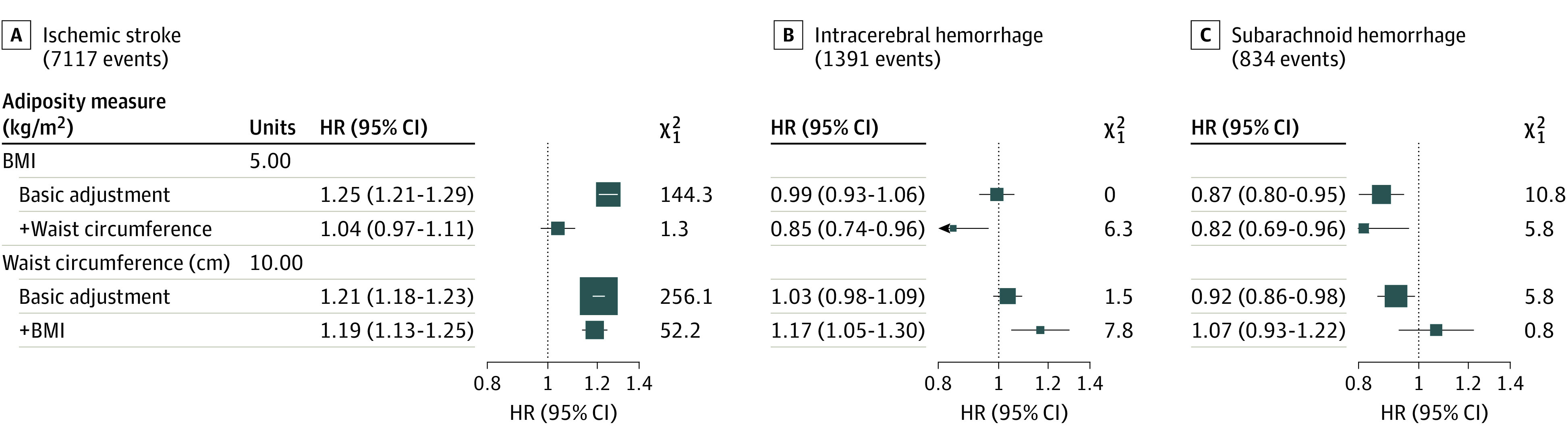
Hazard Ratios (HRs) and 95% CIs per Specified Unit Increase in Adiposity Measures After Baseline and Mutual Adjustment for Other Adiposity Measures, by Stroke Type Analyses adjusted for age, ethnicity, Townsend deprivation, education, smoking status, and alcohol intake. HRs are plotted as squares, with the size of each square proportional to the amount of statistical information. Horizontal lines represent 95% CIs. BMI with ischaemic stroke is only for the linear portion of BMI>25. BMI indicates body mass index.

After mutual adjustment for BMI and waist circumference, BMI was no longer associated with ischemic stroke (HR, 1.04; 95% CI, 0.97-1.11), developed an inverse association with ICH (HR, 0.85; 95% CI, 0.74-0.96), and maintained an inverse association with SAH (HR, 0.82; 95% CI, 0.69-0.96). Waist circumference maintained a positive association with ischemic stroke (HR, 1.19; 95% CI, 1.13-1.25), developed a positive association with ICH (HR, 1.17; 95% CI, 1.05-1.30), and was no longer associated with SAH (HR, 1.07; 95% CI, 0.93-1.22) (Figure 3 and eFigure 2 in the Supplement 1).

In sensitivity analyses, the associations of waist-hip ratio with stroke types were similar to that of waist circumference (eFigure 3 in the Supplement 1). The associations with adiposity measures and each of the stroke types were not materially altered after excluding the first 5 years of follow-up (eFigure 4 in the Supplement 1) or excluding participants with a history of other chronic diseases (eFigure 5 in the Supplement 1).

## Discussion

In this large prospective cohort study of UK adults, in unadjusted analysis, BMI and waist circumference were associated with ischemic stroke, not associated with ICH, and inversely associated with SAH. After mutual adjustment of associations for each adiposity measure, there were independent and contrasting outcomes of BMI and waist circumference with risk of each stroke type: waist circumference was positively associated with ischemic stroke; BMI was inversely associated with SAH; and BMI was inversely associated with ICH, while waist circumference was positively associated with ICH. Although several intermediate factors of the associations of adiposity with stroke types were adjusted for, none accounted for the inverse associations with BMI, indicating that some (as yet unestablished) adverse correlate of low BMI is associated with an increased risk of ICH and SAH.

It is well established that higher levels of adiposity are positively associated with ischemic stroke.^5,7^ However, the present study further demonstrated that central adiposity is chiefly responsible for the increased risk of ischemic stroke rather than general adiposity. The independent positive association of waist circumference with ischemic stroke may be attributed to measures of central adiposity being markers of visceral adipose tissue, which is considered to be particularly atherogenic.^15^ Research on large-scale epidemiological studies with measurements on regional fat depots, such as visceral fat and subcutaneous android fat, are needed to elucidate these mechanisms in more detail.

Evidence regarding associations of BMI with ICH are limited and conflicting.^7^ In a large prospective study^6^ of Chinese adults, BMI was unrelated to ICH at less than 25, and there was a slight positive association at levels greater than 25. In contrast, the present study identified no association with ICH throughout the range of BMI. Notably, a study among 1 million UK women demonstrated an inverse association between BMI and ICH.^7^ There was weak evidence of an inverse association among women in the present study, and possible sex-specific outcomes of BMI on ICH need further investigation.^16^ A meta-analysis,^7^ which includes some of the aforementioned studies, found that BMI was inversely associated with hemorrhagic stroke in European, North American, and Australian populations while a positive association was detected in Asian populations. This disparity could be attributed to artifacts from greater misclassification between stroke types in Asian populations, or real differences in the epidemiology of ICH in these populations.^1^

No identified studies assessed the independent association of BMI with ICH by mutually adjusting for measures of central adiposity. In the present study, BMI developed an inverse association with ICH after further adjustment for waist circumference. This suggests that, at a given level of central adiposity, some adverse correlate of low BMI is associated with an increased risk of ICH, or, equivalently, some protective correlate of high BMI is associated with reduced risk of ICH. By contrast, the null association with waist circumference in the present study became positive following further adjustment for BMI. A study among Chinese adults found little association of waist circumference and ICH risk at lower levels of waist circumference, and slight positive associations at higher waist circumference, but the analyses did not further adjust for BMI.^6^ By assessing the independent relevance of adiposity measures, the present study contributes to the understanding of the specific mechanisms of the different adiposity measures.

Due to the lower incidence of SAH, detecting reliable associations between adiposity measures and SAH is challenging.^6^ However, the present study accrued 834 incident SAH events and demonstrated that BMI was inversely associated with SAH before and after mutual adjustment for central adiposity. By contrast, the inverse association of waist circumference with SAH became null after further adjustment for BMI, suggesting that central adiposity plays a minimal role in SAH risk, independently of general adiposity. Consistent with the present findings, a study among UK women with approximately 2500 SAH events also demonstrated an inverse association of BMI with SAH.^7^ The biological mechanisms underpinning the inverse association between BMI and SAH are not fully understood and require further investigation.^17^ However, aneurysmal rupture, which accounts for approximately 85% of SAHs, has also demonstrated an inverse association with BMI.^17,18^

In mediation analyses, the positive association of general and central adiposity measures with ischemic stroke attenuated by approximately 80% after adjusting for well-established traditional cardiovascular risk factors such as blood pressure, blood lipids, and HbA_1c_. However, besides SBP, traditional cardiovascular factors associated with risk played less of a role in associations between adiposity and ICH. The inverse association between BMI and ICH after adjusting for SBP suggests that some protective correlate of higher BMI potentially offsets the adverse outcome of BMI on SBP, which needs further exploration. Notably, none of the intermediate factors included in the analyses of the present report affected the associations between adiposity measures and SAH, and, therefore, identification of novel factors associated with risk for SAH are needed. The different role of adiposity-related intermediate factors by stroke type supports the distinctly different pathophysiology of stroke types^8^ and highlights the potential for targeted cardiovascular risk factor modification.

### Strengths and Limitations

A key strength of the present study was that it was one of the largest studies to have neuroimaging-confirmed stroke diagnoses, which enabled associations to be determined by stroke types and reduced outcome misclassification. Furthermore, high event rates enabled more reliable associations, particularly for ICH and SAH. Assessing the independent associations of general and central adiposity revealed the specific adiposity measure chiefly responsible for the increased risk of different stroke types. Furthermore, the potential mechanisms underpinning the associations between adiposity measures and stroke types were better understood by evaluating the role of potential intermediate factors associated with risk. The risk of reverse causality was reduced with the prospective study design, excluding those with prior stroke, and further excluding participants with chronic diseases in sensitivity analyses. Moreover, resurvey measurements of adiposity measures enabled correction for regression dilution bias and determined associations by usual levels of adiposity measures which reduced the likelihood of underestimated associations.^19^

Limitations of the present study included not assessing associations by stroke subtypes, which is important as there is some evidence that the associations with other major cardiovascular factors associated with risk may vary by stroke subtype. For example, lacunar ischemic strokes and deep ICHs may be more positively associated with blood pressure than cortical ischemic strokes and lobar ICHs.^20,21,22,23^ However, reliable classification of subtypes of ischemic and hemorrhage stroke are not currently available in UK Biobank; more detailed phenotyping of UK Biobank stroke events would be valuable to future studies. Furthermore, as the majority of the study population were White, there was little power to assess the association in other racial groups, which may have different distributions of body fat^24^ and cardiovascular risk factors.^25^ Lastly, the potential for residual confounding cannot be excluded in observational studies, but future work could further assess the causality of these associations using genetic techniques such as Mendelian randomization.

## Conclusions

In this large cohort study of UK adults, there were independent and contrasting associations of BMI and waist circumference with stroke types. This study suggests the importance of studying body fat distribution to stroke risk, with greater central adiposity a factor associated with risk for ischemic stroke, less general adiposity a factor associated with risk for SAH, and a combination of both factors associated with risk for ICH. Several intermediate factors for the associations of adiposity with stroke types were identified, but none explained the inverse associations with BMI. This suggests that some adverse correlate of low BMI may be associated with an increased risk of ICH and SAH—or, equivalently, some protective correlate of high BMI may be associated with reduced risk of these stroke types—and this warrants further investigation.

## Notes

Supplement 1. **eFigure 1. **Hazard Ratios (95% CIs) per Specified Unit Increase in Adiposity Measures by Stroke Type, Stratified by Baseline Age and Sex **eFigure 2. **Association of Adiposity Measures After Baseline and Mutual Adjustment With Stroke Type **eFigure 3. **Association of Adiposity Measures (Including Waist-Hip Ratio) After Baseline and Mutual Adjustment for Other Adiposity Measures, by Stroke Type **eFigure 4. **Associations of Adiposity Measures With Stroke Type After Excluding the First 5 Years of Follow-up **eFigure 5. **Associations of Adiposity Measures With Stroke Type After Excluding Participants With Chronic Disease

Supplement 2.
Data Sharing Statement
