# Supplementary material for: Adiposity, Body Fat Distribution, and Risk of Major Stroke Types Among Adults in the United Kingdom
Source: JAMA Netw Open. 2022 Dec 14;5(12):e2246613. doi: 10.1001/jamanetworkopen.2022.46613 (PMC9856404; doi:10.1001/jamanetworkopen.2022.46613)
Supplement: Supplement 1. — eFigure 1. Hazard Ratios (95% CIs) per Specified Unit Increase in Adiposity Measures by Stroke Type, Stratified by Baseline Age and Sex eFigure 2. Association of Adiposity Measures After Baseline and Mutual Adjustment With Stroke Type eFigure 3. Association of Adiposity Measures (Including Waist-Hip Ratio) After Baseline and Mutual Adjustment for Other Adiposity Measures, by Stroke Type eFigure 4. Associations of Adiposity Measures With Stroke Type After Excluding the First 5 Years of Follow-up eFigure 5. Associations of Adiposity Measures With Stroke Type After Excluding Participants With Chronic Disease [file jamanetwopen-e2246613-s001.pdf]

Supplemental Online Content

Pillay P, Lewington S, Taylor H, Lacey B, Carter J. Adiposity, body fat distribution, and risk of major stroke types among adults in the United Kingdom. *JAMA Netw Open*. 2022;5(12):e2246613. doi:10.1001/jamanetworkopen.2022.46613

eFigure 1. Hazard Ratios (95% CIs) per Specified Unit Increase in Adiposity Measures by Stroke Type, Stratified by Baseline Age and Sex

eFigure 2. Association of Adiposity Measures After Baseline and Mutual Adjustment With Stroke Type

eFigure 3. Association of Adiposity Measures (Including Waist-Hip Ratio) After Baseline and Mutual Adjustment for Other Adiposity Measures, by Stroke Type

eFigure 4. Associations of Adiposity Measures With Stroke Type After Excluding the First 5 Years of Follow-Up

eFigure 5. Associations of Adiposity Measures With Stroke Type After Excluding Participants With Chronic Disease

This supplemental material has been provided by the authors to give readers additional information about their work.

© 2022 Pillay P et al. *JAMA Network Open.*

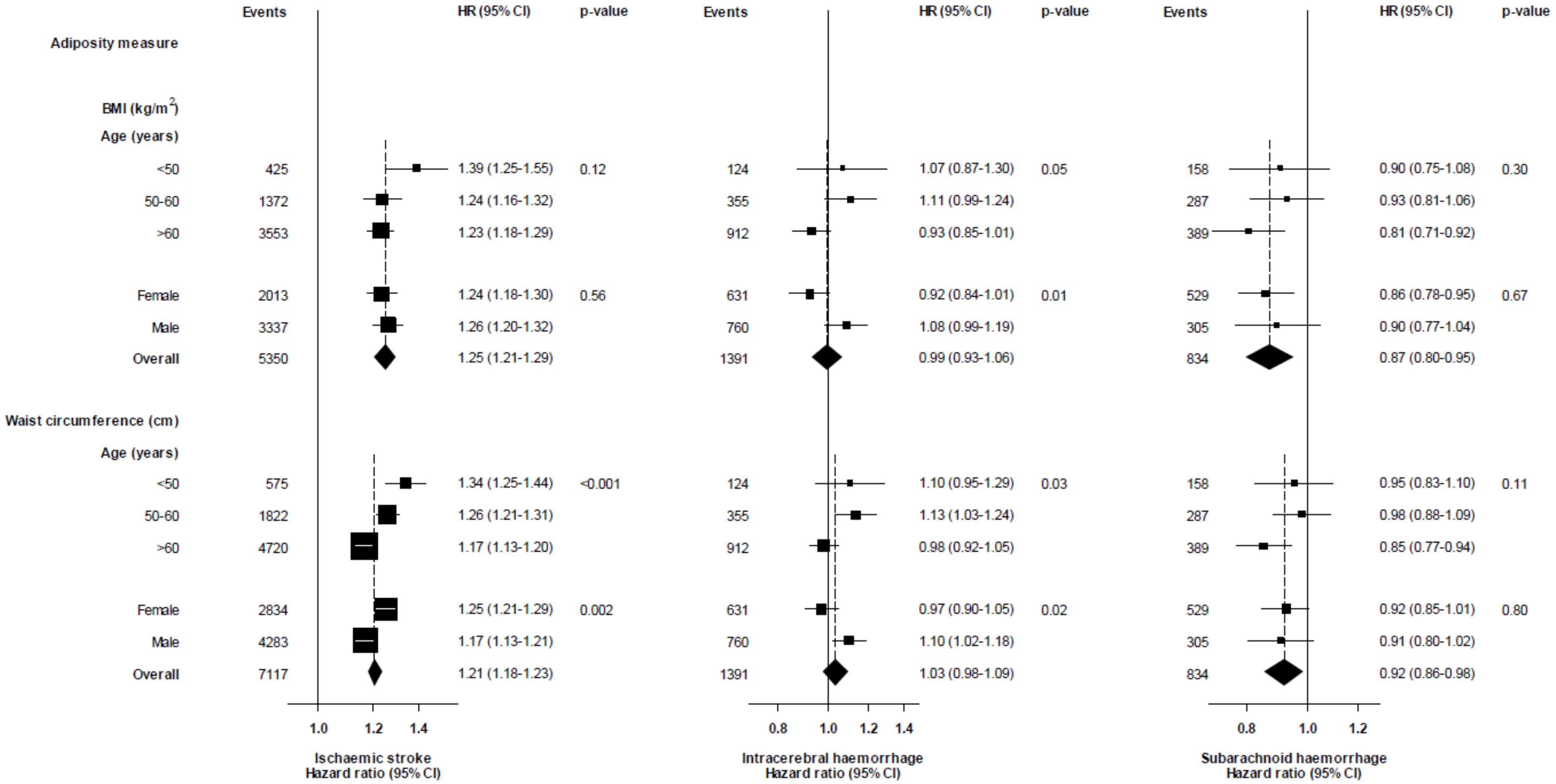

eFigure 1. Hazard Ratios (95% CIs) per Specified Unit Increase in Adiposity Measures by Stroke Type, Stratified by Baseline Age and Sex

Analyses adjusted for age, ethnicity, Townsend deprivation, education, smoking status, and alcohol intake, Group-specific estimates are plotted as squares, with the size of each square proportional to the amount of statistical information. Vertical lines represent group-specific 95% confidence intervals (CIs). BMI with ischaemic stroke is limited to BMI≥25 kg/m^2.

© 2022 Pillay P et al. *JAMA Network Open.*

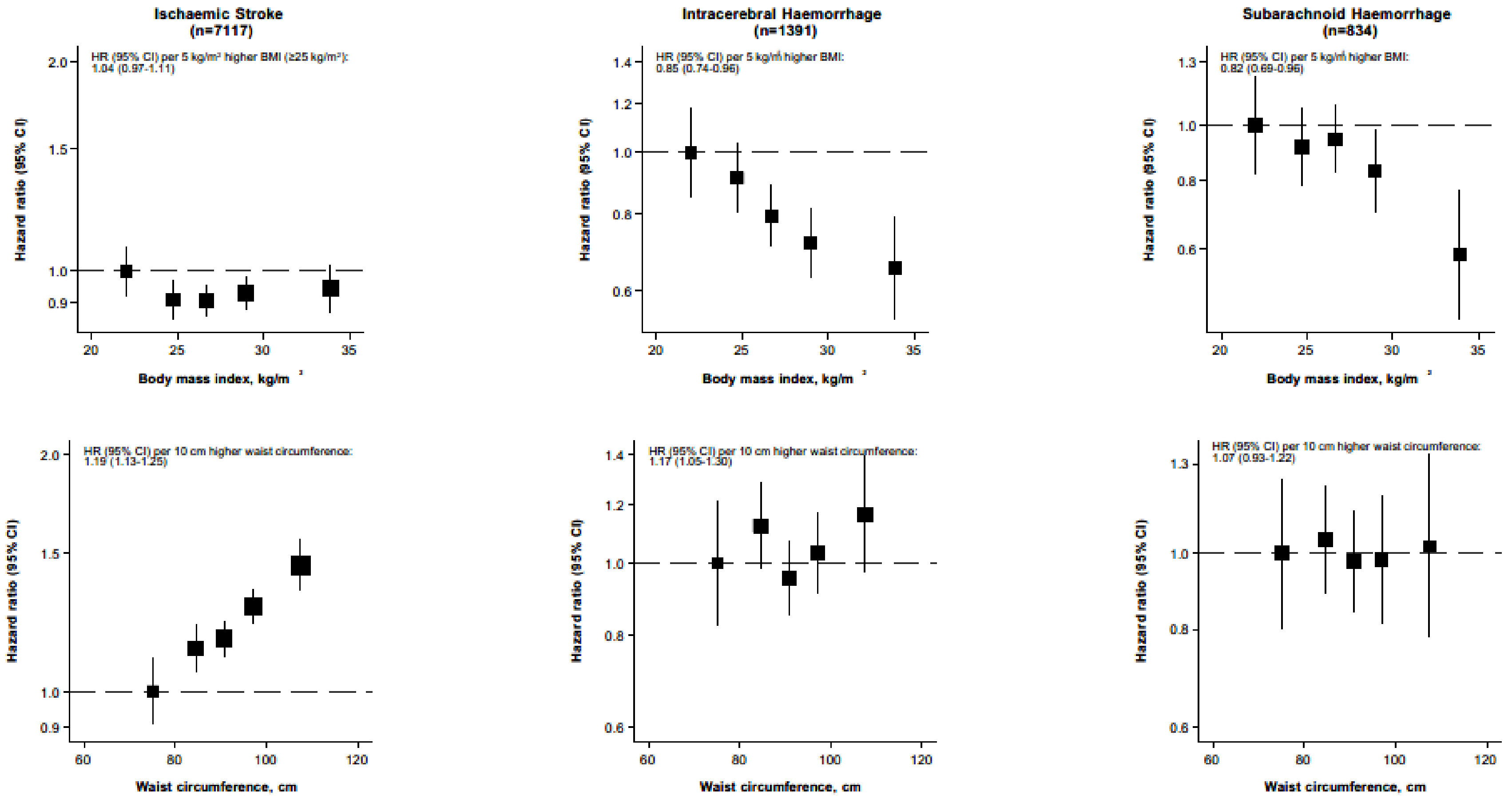

eFigure 2. Association of Adiposity Measures After Baseline and Mutual Adjustment With Stroke Type

BMI, body mass index. Analyses adjusted for age, ethnicity, Townsend deprivation, education, smoking status, and alcohol intake, and wither BMI or waist circumference. Hazard ratios (HRs) are plotted as squares, with the size of each square proportional to the amount of statistical information. The linear trend for BMI with ischaemic stroke is limited to BMI≥25 kg/m^2.

© 2022 Pillay P et al. *JAMA Network Open.*

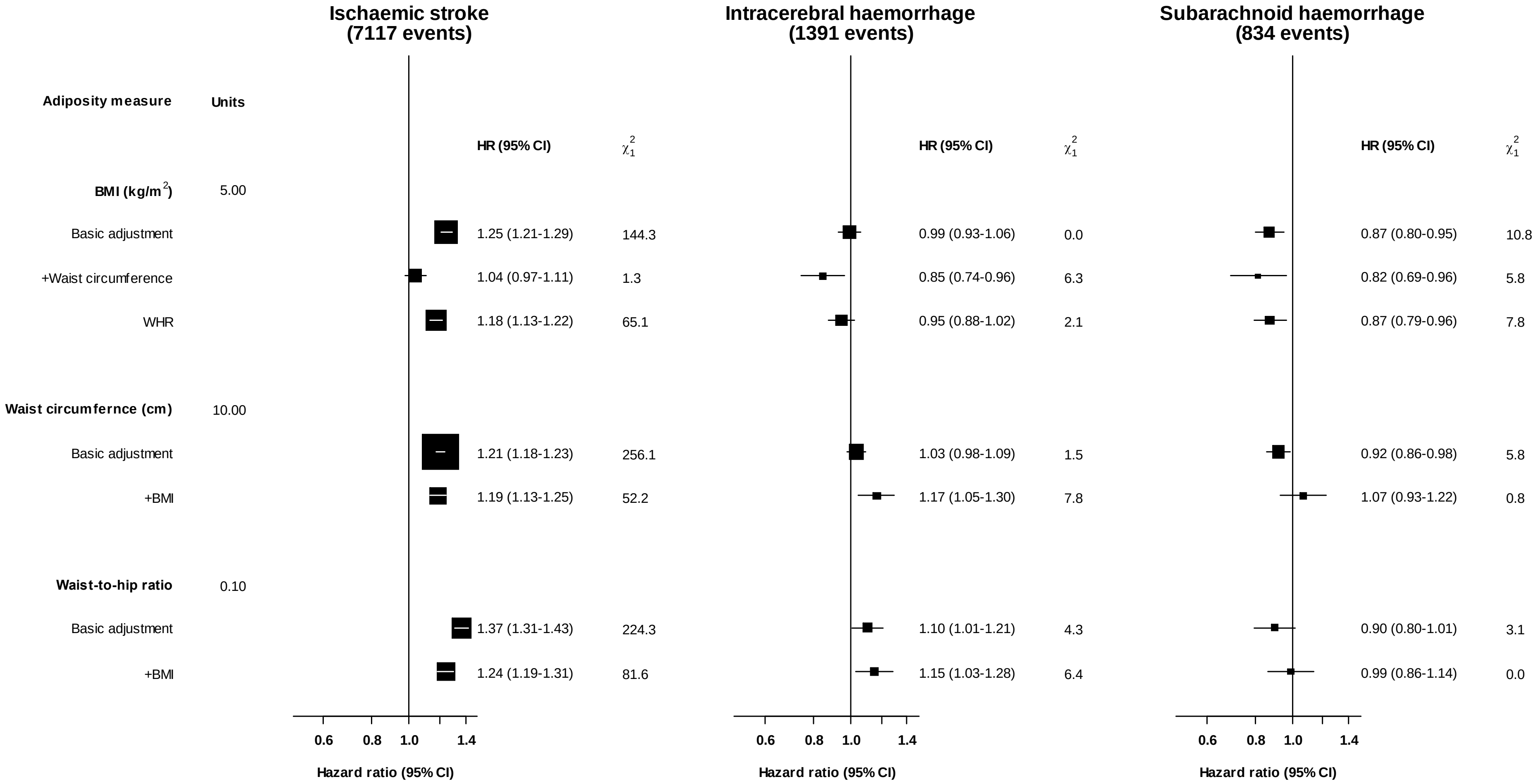

eFigure 3. Association of Adiposity Measures (Including Waist-Hip Ratio) After Baseline and Mutual Adjustment for Other Adiposity Measures, by Stroke Type

BMI, body mass index; WHR, waist-hip ratio. Analyses adjusted for age, ethnicity, Townsend deprivation, education, smoking status, and alcohol intake, Hazard ratios (HRs) are plotted as squares, with the size of each square proportional to the amount of statistical information. Horizontal lines represent 95% confidence intervals (CIs). BMI with ischaemic stroke is limited to BMI≥25 kg/m^2.

© 2022 Pillay P et al. *JAMA Network Open.*

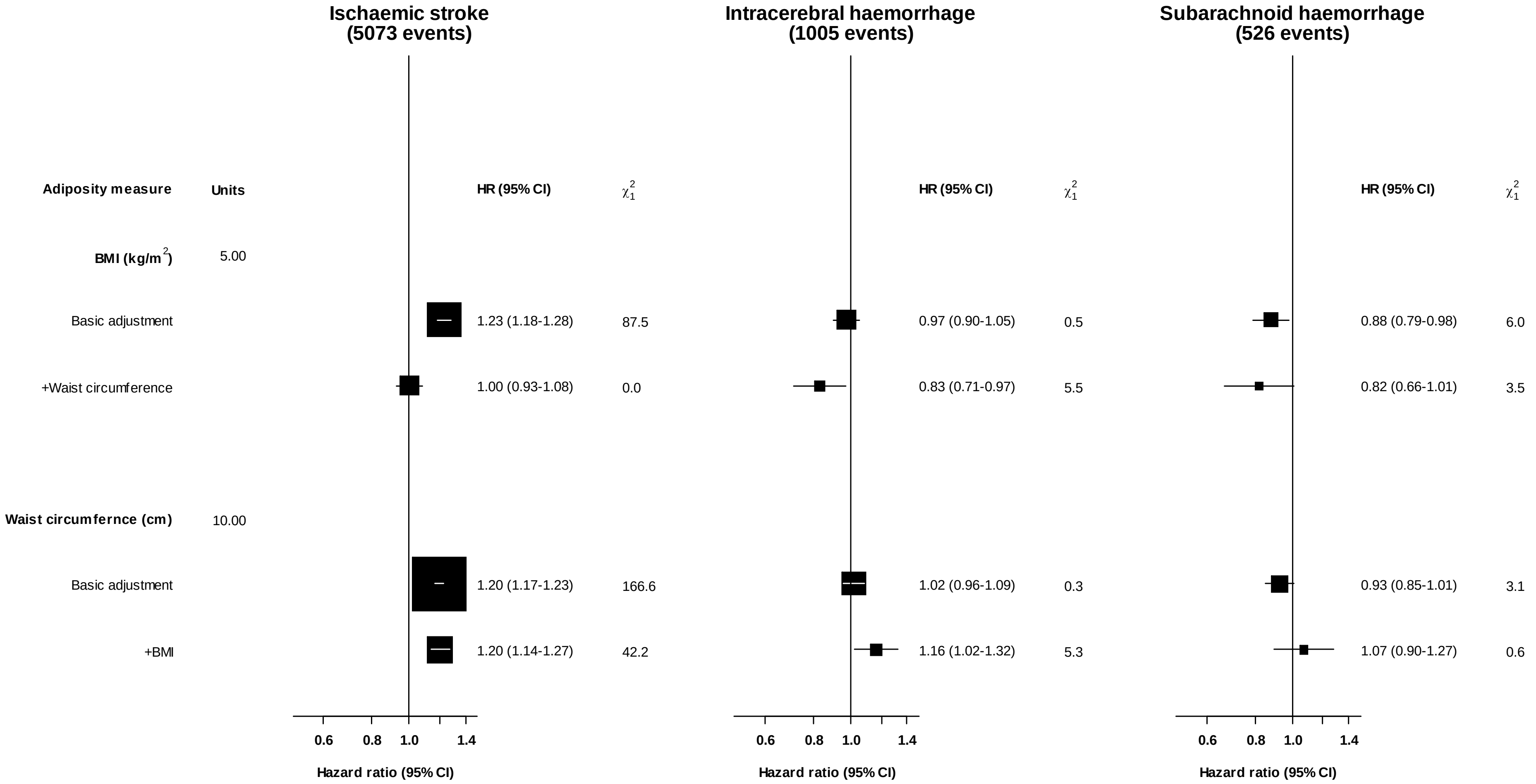

eFigure 4. Associations of Adiposity Measures With Stroke Type After Excluding the First 5 Years of Follow-Up

BMI, body mass index. Analyses adjusted for age, ethnicity, Townsend deprivation, education, smoking status, and alcohol intake, Hazard ratios (HRs) are plotted as squares, with the size of each square proportional to the amount of statistical information. Horizontal lines represent 95% confidence intervals (CIs). BMI with ischaemic stroke is limited to BMI≥25 kg/m^2.

© 2022 Pillay P et al. *JAMA Network Open.*

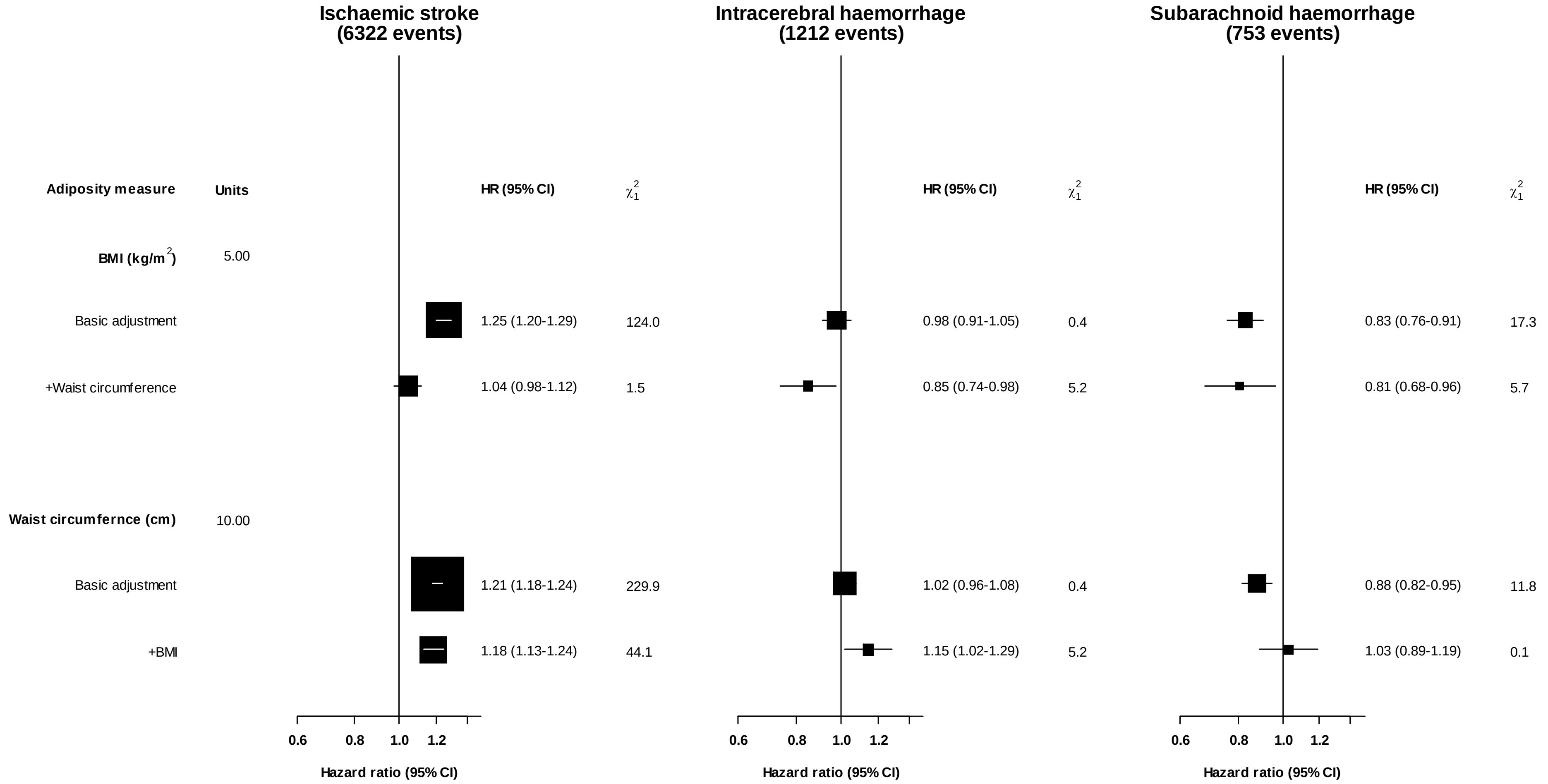

eFigure 5. Associations of Adiposity Measures With Stroke Type After Excluding Participants With Chronic Disease

BMI, body mass index. Those with a history of ischaemic heart disease, myocardial infarction, cancer, emphysema, diabetes, congestive cardiac failure, chronic kidney disease, or chronic liver disease excluded. Analyses adjusted for age, ethnicity, Townsend deprivation, education, smoking status, and alcohol intake. Hazard ratios (HRs) are plotted as squares, with the size of each square proportional to the amount of statistical information. Horizontal lines represent 95% confidence intervals (CIs). BMI with ischaemic stroke is limited to BMI≥25 kg/m^2.

© 2022 Pillay P et al. *JAMA Network Open.*
