# Supplementary material for: Adiposity, Body Fat Distribution, and Risk of Major Stroke Types Among Adults in the United Kingdom
Source: JAMA Netw Open. 2022 Dec 14;5(12):e2246613. doi: 10.1001/jamanetworkopen.2022.46613 (PMC9856404; doi:10.1001/jamanetworkopen.2022.46613)
Supplement: Supplement 2. — Data Sharing Statement [file jamanetwopen-e2246613-s002.pdf]

Data Sharing Statement

Pillay. Adiposity, Body Fat Distribution, and Risk of Major Stroke Types Among Adults in the United Kingdom. *JAMA Netw Open*. Published December 14, 2022.
doi:10.1001/jamanetworkopen.2022.46613

Data
Data available: Yes
Data types: Deidentified participant data
How to access data: All results from this analysis are returned to UK Biobank within 6 months of publication, at which point they can be made available to other researchers upon reasonable request. UK Biobank is an open access resource, and researchers can apply to use the dataset at http://ukbiobank.ac.uk/register-apply/.
When available: With publication

Supporting Documents
Document types: None

Additional Information
Who can access the data: See description above.
Types of analyses: See description above.
Mechanisms of data availability: See description above.
Any additional restrictions: None
